# Impact of COVID-19 Pandemic on the Length of Hospital Stay in Hip Fracture Patients: A Single Centre Study

**DOI:** 10.7759/cureus.27328

**Published:** 2022-07-27

**Authors:** Bakhat Yawar, Jennifer Salmon, Aoife McSorley, Seanna Meehan, Callum Auld, Hassan Abdulrahman, Mohammad Noah Khan, Ammal Imran Qureshi, Ryan Flynn, Ivan Neely, Ali Yawar, Ayeisha Asim, Sami Mustafa, Andrew McAdam, Adriana Sapumohotti, Eimhear Duffy, Hushil Sandhu, Brian Hanratty

**Affiliations:** 1 General Surgery, The Western Trust Health and Social Care (Northern Ireland), Londonderry, GBR; 2 General Surgery, The Western Trust Health and Social Care (Northern Ireland) Altnagelvin Area Hospital, Londonderry, GBR; 3 Trauma and Orthopaedics, Royal Victoria Hospital, Belfast, GBR; 4 Surgery, Worthing Hospital, Southampton, GBR; 5 Trauma and Orthopaedics, The Western Trust Health and Social Care (Northern Ireland), Londonderry, GBR; 6 Geriatrics, The Western Trust Health and Social Care (Northern Ireland), Londonderry, GBR; 7 Urology, The Western Trust Health and Social Care (Northern Ireland), Londonderry, GBR

**Keywords:** economic burden of healthcare, recovery after hip fracture, lenght of hospitalization, frailty hip fracture, geriatric hip fracture

## Abstract

Background

Hip fracture is a debilitating injury, especially in older individuals, which is associated with significant morbidity and mortality. In recent decades, there has been a great focus on early rehabilitation and discharge after hip fractures. The aim of such efforts is to minimize the financial and clinical burden of this condition. We conducted our study during the COVID-19 pandemic and compared the length of hospital stay (LOS) in 2020 to the LOS in 2019. Additionally, we studied the factors which may impact the LOS, such as premorbid status according to established scoring systems, the type of fracture, an operation performed, and time to surgery.

Methods

We collected the data regarding the length of stay (in days) for all hip fracture patients admitted to our unit from 1^st^ January 2019 until 31^st^ December 2020. We then compared the mean LOS for both years using the t-test. We calculated the Nottingham Hip Fracture Score (NHFS) and American Society of Anaesthesiologists (ASA) scores for patients admitted in 2020 and calculated the correlation between increasing values of these scores and the LOS. We also compared the mean LOS for patients admitted in 2020 based on the type of fracture and type of management. We studied the correlation between the time to surgery and the LOS for patients admitted in 2020.

Results

Three hundred and eighty-eight patients were admitted with hip fractures in 2020, and 452 were admitted in 2019. LOS in 2020 was significantly lower (23.39 days) compared to 2019 (31.36 days) with p<0.01. While evaluating data from 2019, it was noted that there was a small positive correlation between LOS and NHFS (r=0.231, p<0.001) and LOS and ASA (r=0.18, p<0.001). The mean LOS for intracapsular fractures was noted to be lower than that of extracapsular fractures, but this was not statistically significant (p=0.17). An ANOVA test showed that the mean LOS for patients undergoing hemiarthroplasty, dynamic hip screws (DHS), and intramedullary nails (IMN) was significantly longer than for patients managed with total hip replacement or patients managed non-operatively (F=3.551, p<0.01).

Conclusion

Hip fracture patients admitted to our department were discharged quicker during the first year of the COVID-19 pandemic. The LOS for hip fractures increases with an increase in their NHFS or ASA scores. Extracapsular and intracapsular fractures lead to roughly the same periods of inpatient stay. Patients undergoing hemiarthroplasty, DHS, or IMN stay longer in the hospital compared to other treatment modalities.

## Introduction

Hip fracture in elderly patients is a devastating injury with an annual incidence reported between 70,000 to 75,000 in the UK [1]. The National Hip Fracture Database (NHFD) [2] aims to collect and report data on hip fracture patients and collates data regarding performance indicators such as prompt orthogeriatric review and prompt surgery as per the Best Practice Tariff (BPT) [3], postoperative confusion, destination of discharge, etc. The NHFD reported the mean trust length of stay (LOS) as 14.5 days in May 2020 overall in the UK, whereas the trust LOS in 2019 was 19.3 days [2].

Management of hip fractures carries a significant economic burden on the NHS. Previous estimates suggest that index hip fractures lead to hospital costs of approximately £14,163 in the first year after sustaining the injury [4]. The total cost of management of hip fractures in the UK per annum is reported to be £2-£3 billion [4,5]. Hip fractures have been reported to be the costliest osteoporotic fractures to manage [6].

These staggering costs have led to the development of several guidelines for the management of hip fractures with great emphasis on early mobilisation and rehabilitation, which leads to early recovery and lowers the overall expenditure. These guidelines include the National Institute of Clinical Excellence (NICE) guidelines for the management of hip fractures [7], the Best Practice Tariff [3], the Care of Patients with Fragility Fractures Blue Book by the British Geriatrics Society [8], and the British Orthopaedics Association Standards for Trauma (BOAST) guideline for the management of frail or elderly orthopaedics patient [9]. These guidelines form the cornerstone for the management of hip fractures in our hospital trust as well.

The primary aim of our study was to compare the LOS in our trust during the year 2020 (which was the first year of the COVID-19 pandemic) to the LOS in 2019 and evaluate if there was any difference in the mean LOS. Secondarily, we studied some factors which may impact the LOS for the patients admitted during 2020. These included the American Society of Anaesthesiologists (ASA) score and the Nottingham Hip Fracture Score (NHFS). Increasing values of both these scoring systems denote higher morbidity. In addition, we studied whether the type of fracture or the type of management offered to patients had any impact on the LOS. We also studied if the time from admission to surgery had any impact on the LOS as well.

## Materials and methods

Study design

A retrospective cohort study was conducted on patients admitted from 1^st ^January 2019 until 31^st^ December 2020 to Altnagelvin Area Hospital in Derry/Londonderry, Northern Ireland. We registered the project with our local Audit and Quality Improvement Department in the trust. A formal ethics approval or institutional review board approval was not needed for this study as per guidelines from National Health Service Health Research Authority (NHS HRA) regulations.

Inclusion and exclusion criteria

For the comparison of LOS between 2019 and 2020, we included all patients admitted during this period of two years with hip fractures. To ascertain the impact of various factors on LOS, we included only patients admitted in 2020.

After a careful review of the records submitted to the National Hip Fracture Database (NHFD), we excluded all non-hip femoral fractures such as periprosthetic, distal femoral, and femoral shaft fractures. We excluded patients below 18 years of age as well.

Data collection

A database for all patients admitted with hip fractures was obtained from specialist nurses. This database included data on patient demographics, abbreviated mental test score (AMTS), usual place of residence, and co-morbidities. The ASA score was available from the database on all patients. NHFS was calculated after reviewing patient records in Northern Ireland Electronic Care Record (NIECR) system.

Data variables collected

Length of stay (LOS) in the trust facilities was noted for all hip fracture patients admitted during the two-year study period. To determine the impact of different factors, we collected demographic data on all patients admitted in 2020 to formulate the Nottingham Hip Fracture Score (NHFS). This included the age, gender, AMTS, place of usual residence, and co-morbidities. Co-morbidities were noted as cardiovascular (including ischaemic heart disease, congestive heart failure, atrial fibrillation, previous myocardial infarction, hypertension, cardiomyopathy, bundle branch block, peripheral vascular disease), respiratory (including asthma, chronic obstructive pulmonary disease, bronchiectasis, lung fibrosis, etc.), renal (including nephropathy, chronic kidney disease, etc.) and endocrine (including diabetes, Addison’s disease, Cushing’s disease, hypothyroidism, hyperthyroidism, etc.). We did not report the co-morbidities separately as these were used to only calculate the NHFS for all the patients. We also reviewed the NIECR to review the haemoglobin level and the presence of active malignancy (apart from squamous cell carcinoma and basal cell carcinoma) to calculate the NHFS, which ranges from 0-10 [10]. The ASA score was available from the data already reported to the National Hip Fracture Database (NHFD) and ranged from 0-5 for our patients. Fractures were classified as extracapsular (which include intertrochanteric and subtrochanteric hip fractures) and intracapsular (which include subcapital, transcervical, and basicervical hip fractures). Time from admission to surgery was noted in hours. Management offered to patients included non-operative management and operative management (which includes cannulated screws, dynamic hip screw, hemiarthroplasty, total hip replacement, and intramedullary nail).

Data analysis

Firstly, we compared the mean trust LOS for patients admitted from 1^st^ January 2019 until 31^st^ December 2019 with the mean trust LOS for patients admitted from 1^st^ January 2020 until 31^st^ December 2020 using an unpaired t-test. Spearman rank order correlation test was then used to calculate the correlation between the ASA score and the trust LOS for patients admitted from 1^st ^January 2020 until 31^st^ December 2020. Spearman rank order correlation was also used to calculate the correlation of NHFS as well as time to surgery with the trust LOS for patients admitted in 2020. We used the Spearman rank order correlation test to find an association between delay to surgery in hours and trust LOS. We also conducted a t-test to compare the means of the patients who underwent surgery within 48 hours of admission to those who underwent surgery after 48 hours. We used the analysis of variance (ANOVA) test to determine if there was any difference between the mean LOS of patients managed with different management modalities in 2020.

## Results

Primary aim

Four hundred and fifty-two patients were admitted with hip fractures to our unit in 2019, while 388 patients were admitted in 2020. An unpaired t-test was used to compare the mean trust LOS in 2019 to the mean trust LOS in 2020. We noted that the mean LOS in 2020 was much lower than in 2019. This difference was statistically significant (p<0.0001). Table 1 below shows the details of LOS in 2019 and 2020 in more detail.

**Table 1:** Details of LOS in 2019 and 2020 LOS - length of stay

Group		2019	2020
LOS (days)	Mean	31.36	23.39
	Standard deviation	32.41	22.09
	Range	1-367	2-152

Secondary aims

Secondary aims of identifying factors impacting the trust LOS were based on data collected on patients admitted from 1^st ^January 2020 until 31^st^ December 2020. During this period, 388 patients were admitted. Among them, 380 patients were managed operatively.

Baseline Characteristics

Patients admitted with hip fractures were elderly. Intracapsular fractures were more than twice as common as extracapsular fractures, with hip hemiarthroplasty being the commonest operation performed. More than two-thirds of patients were female. Baseline demographic factors are further elaborated in Table 2 below.

**Table 2:** Demographics and baseline characteristics of patients admitted with hip fractures in 2020 AMTS - abbreviated mental test score; DHS - dynamic hip screw; CNS - cannulated hip screws; IMN - intramedullary nail; THR - total hip replacement

Variable	Category	n=388	Value
Age (years)	Mean +/- standard deviation	388	78.19 +/- 11.45
	Median		81
	Range		30-97
Gender	Male	388	120 (30.9%)
	Female		268 (69.1%)
AMTS	Less than 7	388	98 (25.2%)
	7 or above		290 (74.8%)
Usual place of residence	Own home	388	334 (86.1%)
	Institutionalised		54 (13.9%)
Type of Fracture	Extracapsular	388	124 (31.9%)
	Intracapsular		264 (68.1%)
Type of management	Hemiarthroplasty	388	198 (51.0%)
	DHS		94 (24.2%)
	CNS		6 (1.5%)
	IMN		59 (15.3%)
	THR		23 (5.9%)
	Non-operative		8 (2.1%)
Time to surgery	Within 48 hours	380	283 (74.5%)
	After 48 hours		97 (25.5%)

Relation Between NHFS and LOS

**Figure 1:**
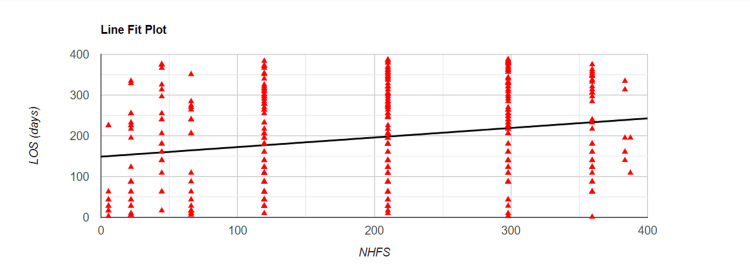
Spearman correlation plot for the association between NHFS and LOS NHFS - Nottingham Hip Fracture Score; LOS - length of stay

We performed the Spearman correlation test to investigate any correlation between the Nottingham Hip Fracture Score (NHFS) and the LOS. We found a weak positive correlation between NHFS and LOS with r= 0.23. This result was statistically significant with p<0.001. As the patient's NHFS increases, so are the chances for prolonged hospital admission. Figure 1 below sheds further light on this correlation.

Relation Between ASA and LOS

**Figure 2:**
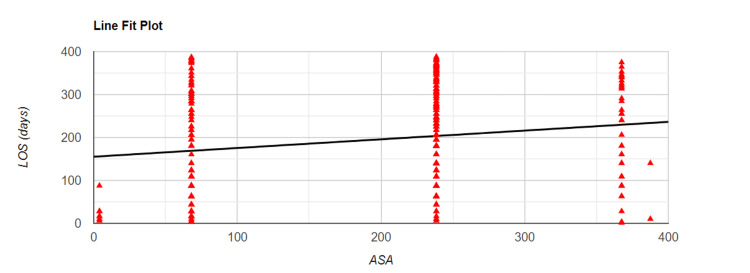
Spearman correlation plot detailing association between ASA score and LOS ASA - American Society of Anaesthesiologists score; LOS - length of stay

We conducted the Spearman correlation test to understand an association between patient ASA scores and trust LOS. We noted a weak positive correlation between ASA scores and LOS with r= 0.18, p<0.001. Figure 2 below details the correlation in graphical form.

Relation Between Time to Surgery and LOS

**Figure 3:**
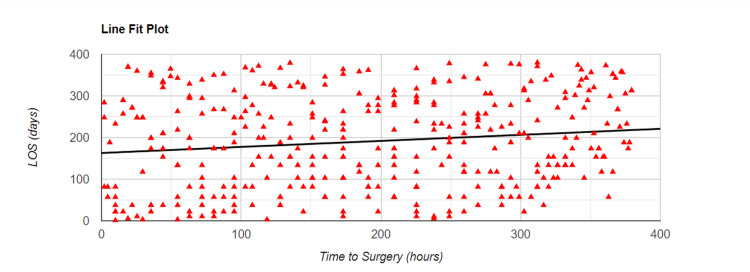
Spearman correlation plot showing the association between time to surgery and LOS LOS - length of stay

Firstly, we noted the time from admission to surgery in hours. Then, we used the Spearman correlation test to understand if there was an association between time to surgery and the LOS. The results were statistically significant with a p-value of 0.005 and showed a weak positive correlation between the two variables with r=0.15. Figure 3 below details the correlation in graphical form.

We also conducted a t-test to compare the means of the patients who underwent surgery within 48 hours of admission to those who underwent surgery after 48 hours. We noted a higher mean LOS for patients who underwent surgery after 48 hours (t=2.28, p=0.023). This is explained further in Table 3 below.

**Table 3:** LOS for early versus late surgery LOS - length of stay

Group		Surgery within 48 hours	Surgery after 48 hours
LOS (days)	Mean	22.21	28.10
	Standard deviation	20.28	26.47
	N	283	97

Type of Fracture and LOS

An unpaired t-test analysis was performed to determine if there was a difference in mean LOS for extracapsular and intracapsular fractures. The difference between the mean LOS of extracapsular and intracapsular fractures was not significant (t=1.39, p=0.17). The mean LOS for the types of fractures is described in Table 4 below.

**Table 4:** LOS for different types of hip fractures LOS - length of stay

Group		Extracapsular	Intracapsular
LOS (days)	Mean	25.65	22.32
	Standard deviation	23.99	21.08
	Range	2-152	1-144

Type of Management and LOS

An analysis of variance (ANOVA) test was conducted to investigate the difference between LOS for different management modalities. The result showed a statistically significant difference with higher LOS noted for patients managed with hemiarthroplasty, DHS, and IMN compared to patients managed with THR and patients managed non-operatively (F=3.55, p=0.004). Priori power for the test was 0.98. Table 5 below explains the LOS for various management options.

**Table 5:** LOS associated with different management options LOS - length of stay; DHS - dynamic hip screw; CNS - cannulated hip screws; THR - total hip replacement; IMN - intramedullary nail

Group		Hemiarthroplasty	DHS	IMN	THR	CNS	Conservative
LOS (days)	Mean	24.49	23.83	27.41	8.57	17.83	7.88
	Standard deviation	20.93	21.31	29.14	5.60	13.86	11.10
	Range	3-144	5-105	6-152	2-24	3-39	1-34

**Figure 4:**
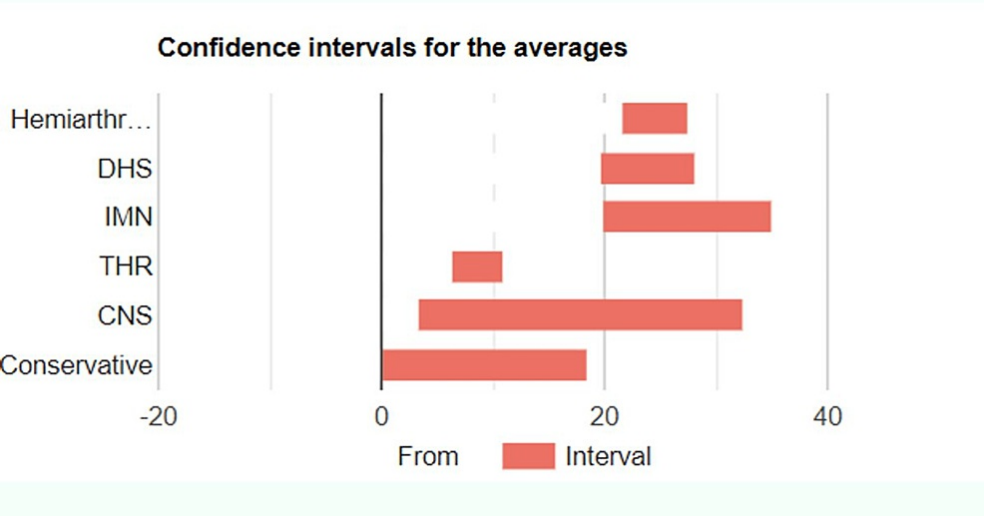
Confidence interval overlaps for LOS with various management options LOS - length of stay; hemiarthr - hemiarthroplasty; DHS - dynamic hip screw; CNS - cannulated hip screw; IMN - intramedullary nail; THR - total hip replacement

Figure 4 below elaborates confidence intervals for mean LOS for different management options.

## Discussion

The World Health Organisation (WHO) declared COVID-19 a pandemic on March 11, 2020, and recommended actions to contain the spread of the virus [10]. After emerging in Wuhan City in China as an epidemic, the disease took all the continents by storm and developed into a true pandemic [11]. The pandemic had profound negative effects on healthcare, business, and the economy [12].

In our study, we noted a significant reduction in the trust LOS in our patients admitted in the first year of COVID-19 compared to the previous year, i.e., 2019. The drop in LOS was noted in other centres in the UK, and this was reflected in the NHFD report in 2021, which reported a mean LOS of 14.5 days in May 2020 compared to 19.7 days in 2019 [2]. Although we noted a reduction in the LOS in 2020, our overall length of stay of 23.39 days was still higher than the mean LOS in the UK. The reason behind this prolonged LOS in our hospital is unclear, but our consultant body reports that historically our hip fracture patients stay longer in hospital compared to the national average. We aim to conduct further studies to ascertain the cause behind this finding.

It has been reported in the past that a higher Nottingham Hip Fracture Score (NHFS), especially with values of seven or above, is associated with prolonged inpatient stay [13]. The Spearman correlation test also found that the LOS after hip sustaining hip fractures increases as the NHFS increases. NHFS was initially devised to predict 30-day mortality in hip fracture patients in 2007 [14,15] and has been validated by further studies for predicting one-year mortality as well [16]. Our findings indicate that the Nottingham Hip Fracture Score can also predict increasing LOS for patients with increasing values of NHFS.

American Society of Anaesthesiologists (ASA) score is a classification system for pre-operative physiological status, which was devised in 1941 and has been revised since that time [17]. With values ranging from one to six, it reliably predicts increasing postoperative morbidity and mortality for all surgical procedures [17,18,19]. It is a simple tool for predicting postoperative complications and is used by anaesthetists across the globe daily [19]. ASA score has been validated in previous studies as a predictor of LOS. It has been previously reported that as the ASA score increases, so does the LOS [20-22]. Our study also demonstrated similar results with increasing LOS as the ASA score increases.

NICE guidelines for hip fracture management recommend surgery on the day of or the day after admission [7], whereas the Best Practice Tariff recommends operation within 36 hours [3]. As the Best Practice Tariff is currently not applicable to Northern Ireland, we defined early surgery in case of hip fractures as within 48 hours of admission to our unit. Previously, it has been reported that early surgery after sustaining hip fractures is associated with a shorter duration of stay in the hospital [22-24]. We noted similar findings from our study. Surgery after 48 hours of admission was associated with a longer LOS. Additionally, as the duration of the delay to surgery increased, so did the LOS.

We noted in our study that the LOS did not vary significantly for extracapsular and intracapsular hip fractures. In addition, we also noted that patients with hip fractures managed with hemiarthroplasty, dynamic hip screws, or intramedullary nails had a longer duration of LOS compared to patients managed conservatively or with total hip replacement. The reason behind these findings is likely because total hip replacement is offered to patients with a good baseline physiological status who are independently mobile before the injury in our hospital, whereas patients managed with hemiarthroplasty, DHS, or IMN are generally frailer. Additionally, patients who were managed conservatively in our hospital were moribund at admission, and for many such patients, the hip fracture was a terminal event. We have not seen much data published on LOS variation by type of fracture or by type of management, and we recommend further in-depth research on these topics.

Our study included a high number of patients, due to which we were able to draw adequate conclusions from our results. However, this was single-centre study, and the guidelines followed in Northern Ireland are sometimes different from those followed in the rest of the United Kingdom, such as the BPT. Therefore, the results from our study may not be generalisable to the rest of the UK. We recommend further multi-centre studies to evaluate the length of stay and economic burden of hip fractures on a national level with further focus on the type of injury and the type of management offered to patients.

## Conclusions

A shorter duration of stay was noted for hip fractures in the year after the start of the COVID-19 pandemic compared to the previous year. Additionally, we found the Nottingham Hip Fracture Score and the ASA score as good predictors of the length of hospital stay after hip fractures. These scoring systems can be used to predict the length of stay for hip fracture patients. We recommend further large-scale studies should be conducted to ascertain if NHFS and ASA can predict costs associated with hip fracture as well. Furthermore, early surgery on the day of or the day after sustaining a hip fracture, as recommended by NICE guidelines, should be expedited in order to ensure a shorter LOS for hip fracture patients.
